# Effectiveness of AbobotulinumtoxinA in Post-stroke Upper Limb Spasticity in Relation to Timing of Treatment

**DOI:** 10.3389/fneur.2020.00104

**Published:** 2020-02-28

**Authors:** Jörg Wissel, Klemens Fheodoroff, Maurits Hoonhorst, Martina Müngersdorf, Philippe Gallien, Niklaus Meier, Jürgen Hamacher, Harald Hefter, Pascal Maisonobe, Manuel Koch

**Affiliations:** ^1^Vivantes Hospital Spandau, Berlin, Germany; ^2^Gailtal-Klinik, Hermagor-Pressegger See, Austria; ^3^Center for Rehabilitation Vogellanden, Zwolle, Netherlands; ^4^Neurologisches Zentrum für Bewegungsstörungen und Diagnostik, Berlin, Germany; ^5^Pôle Saint Hélier, Rennes, France; ^6^Department of Neurology, University Hospital Bern and University of Bern, Bern, Switzerland; ^7^Praxis für Neurochirurgie, Essen, Germany; ^8^Department of Neurology, University of Düsseldorf, Düsseldorf, Germany; ^9^Ipsen Pharma, Boulogne-Billancourt, France; ^10^IPSEN PHARMA GmbH, Munich, Germany

**Keywords:** abobotulinumtoxinA, botulinum toxin, Dysport, spasticity, stroke

## Abstract

**Background:** Recent studies of botulinum toxin for post-stroke spasticity indicate potential benefits of early treatment (i. e., first 6 months) in terms of developing hypertonicity, pain and passive function limitations. This non-interventional, longitudinal study aimed to assess the impact of disease duration on the effectiveness of abobotulinumtoxinA treatment for upper limb spasticity.

**Methods:** The early-BIRD study (NCT01840475) was conducted between February 2013 and 2018 in 43 centers across Germany, France, Austria, Netherlands and Switzerland. Adult patients with post-stroke upper limb spasticity undergoing routine abobotulinumtoxinA treatment were followed for up to four treatment cycles. Patients were categorized by time from stroke event to first botulinum toxin-A treatment in the study (as defined by the 1st and 3rd quartiles time distribution) into early-, medium- and late- start groups. We hypothesized that the early-start group would show a larger benefit (decrease) as assessed by the modified Ashworth scale (MAS, primary endpoint) on elbow plus wrist flexors compared with the late-start group.

**Results:** Of the 303 patients enrolled, 292 (96.4%) received ≥1 treatment and 186 (61.4%) received 4 injection cycles and completed the study. Patients in all groups showed a reduction in MAS scores from baseline over the consecutive injection visits (i.e., at end of each cycle). Although reductions in MAS scores descriptively favored the early treatment group, the difference compared to the late group did not reach statistical significance at the last study visit (ANCOVA: difference in adjusted means of 0.15, *p* = 0.546).

**Conclusions:** In this observational, routine-practice study, patients in all groups displayed a benefit from abobotulinumtoxinA treatment, supporting the effectiveness of treatment for patients at various disease stages. Although the data revealed some trends in favor of early vs. late treatment, we did not find strong evidence for a significant benefit of early vs. late start of treatment in terms of reduction in MAS scores.

## Introduction

A significant percentage of patients develop upper limb spasticity after stroke. In general, upper limb muscles are more affected than lower limb muscles, with the arm being severely affected in about 30% of stroke survivors (1–3). Spasticity interferes with routine task performance, contributes to the development of joint contractures and pain, makes hygiene, and self-care difficult and ultimately has great impact on patient and caregiver quality of life (QoL) (4–7). Spasticity may evolve early in the post-stroke period, with one in five patients developing spasticity within 3 months of the stroke event (8, 9). Some studies have demonstrated muscle tone changes in the affected limbs within just 3 weeks after the stroke event (10–12).

Botulinum neurotoxin A (BoNT-A), including abobotulinumtoxinA (Dysport®, Ipsen Pharma, Wrexham UK), is recommended as a first-line pharmacological treatment option for spasticity (13, 14), but is not typically initiated until spasticity is well-established, and often much later (15). Systematic reviews based on randomized, controlled trial evidence have confirmed that BoNT-A is well-tolerated and effective for the treatment of upper limb spasticity (16, 17). However, to date, most interventional studies have been restricted to patient cohorts with chronic spasticity (i.e., at least 6 months, and an average of 2.5 years post-stroke) (16–18). This limited evidence-base has influenced current guidelines remaining unclear about treatment goals considering different stages and severity of spasticity. AbobotulinumtoxinA is approved for the management of adult upper (and lower) limb spasticity. Recent randomized, placebo-controlled data indicate potential benefits of early treatment with abobotulinumtoxinA in terms of delaying development of hypertonicity, reducing pain and passive function limitations (18–20), and it has further been suggested that early injections may be helpful in preventing contracture development, with potential to unmask active functional improvement (18, 21). Indeed, exploratory analyses of studies of abobotulinumtoxinA in upper limb spasticity management have suggested that the most influential factors predicting goal achievement are previous treatment status (whether the patients were *de novo* or had been previously treated with BoNT-A) and time since spasticity onset as well as the spasticity pattern, and overall injection dose (22).

The aim of the early-BIRD (early Botulinum toxin treatment: Initial and Repeated Documentation) study was to evaluate the real-world effectiveness of abobotulinumtoxinA on the evolution of spasticity in patients with post-stroke upper limb spasticity according to the time from stroke to start of BoNT-A treatment. We hypothesized that patients who start treatment with abobotulinumtoxinA early in their treatment journey will show a larger effect (i.e., reduction in spasticity from baseline) as assessed by the composite sum of the modified Ashworth scale (MAS) at the elbow and wrist flexors when compared to those who start treatment later in their disease course.

## Methods

### Study Setting

The early-BIRD study was an international, multicenter, non-interventional, prospective, longitudinal study conducted in 303 post-stroke survivors undergoing treatment in 43 centers specializing in outpatient spasticity treatment across Germany, France, Austria, Netherlands and Switzerland. The study began in February 2013, recruitment continued until February 2016, and the study completed in February 2018. The study was conducted in compliance with the Declaration of Helsinki, the International Ethical Guidelines for Epidemiological Studies and the International Society for Pharmacoepidemiology (ISPE) Guidelines for Good Pharmacoepidemiology Practices (GPP); it was registered at clinicaltrials.gov as NCT01840475. Ethics approval was obtained from the relevant independent ethics committee at each study center. All patients provided written informed consent for trial participation, including specific consent that they were willing to fill in the QoL questionnaire (EQ-5D-3L) at three visits.

Since this was a non-interventional study, investigators were asked to report adverse events (AEs) to the safety department of the drug manufacturer using the usual local process for such reactions.

### Patients

Patients were recruited on an out-patient basis through the participating specialist centers (BoNT-A clinics, rehabilitation clinics, or neurological practices) where they were undergoing routine assessment and treatment. Investigators recruited all adult patients (aged at least 25 years old) with hemiparesis and clinically relevant post-stroke upper limb spasticity who consented to study participation during a pre-defined time-frame. Eligible patients were either currently being treated with a BoNT-product or considering starting treatment in line with the local prescribing information and usual medical practice. The decision to prescribe abobotulinumtoxinA was made prior to and independently from the decision to enroll the patient in this non-interventional study. Out-of-routine diagnostic or therapeutic interventions were not permitted during this study. Key exclusion criteria included: recurrent stroke, sensitivity to abobotulinumtoxinA, or its excipients, any contraindications as given in the local SmPC for Dysport®, and current participation in an interventional trial.

The maximum number of patients per center was 20. Investigators were permitted to space the inclusions (e.g., inclusion of 1 patient after every 2, or 3, etc. patients) but had to follow the same recruitment frequency until achievement of the recruitment target.

### Assessments

Study data collected as part of routine medical care were captured using an electronic Case Report Form (eCRF). Aside from the EQ-5D-3L which was self-completed by the patients (with or without caregiver assistance), investigators were only required to record outcome assessments they routinely perform in their clinical practice. Thus, some sites did not complete all sections contained within the eCRF. Patients were followed for a maximum of 4 routine abobotulinumtoxinA treatment cycles. The timing of assessments was in accordance with routine medical practice for the investigator. Other than this, no specific instructions on the timing of treatment were given in the study protocol.

The primary measurement of effectiveness was the modified Ashworth Scale (23) (composite sum of elbow and wrist flexors; MAS_EWF_) at the end of treatment cycle 4 (visit 5) or last study visit. The composite MAS_EWF_ is the sum of the MAS measured at the elbow and at the wrist, which was chosen for this routine practice study because is easier to perform than determining a primary targeted muscle group. Other routine assessments included demographics and relevant medical history, date of stroke event, use of physical and occupational therapy, pattern of upper limb spasticity involvement (24), passive and active Range of Motion (PROM and AROM) assessments, pain assessment [on a visual analog scale [VAS] at rest], and treatment satisfaction, as well as injection details (dose, muscles injected etc.). In addition, many specialist centers routinely use a goal setting approach, including Goal Attainment Scaling (GAS) to assess effectiveness of the treatment (25, 26). Investigators negotiated and agreed the main treatment goal(s) with the patient at the baseline visit. As previously suggested (27), goals were categorized under the following six domains: improvement of mobility, pain reduction, ease of care and hygiene, support and ease of physiotherapy (PT) and/or occupational therapy (OT), functional improvement (with definition of individual functional goal) and other (to be specified). Goal attainment was rated as “fully achieved,” “partly achieved,” or “not achieved” at each visit. Investigators were asked to report adverse drug reactions directly to the safety department of the study sponsor.

### Statistical Analyses

The study population included all patients who received ≥1 injection of abobotulinumtoxinA and had ≥1 valid MAS measurement post-baseline. For the primary effectiveness endpoint, patients were categorized into sub-groups (early-start, medium-start or late-state) according to the first and third quartiles time distribution (first quartile = early group; final quartile = late group) since the stroke event until start of BoNT treatment.

The primary effectiveness assessment (MAS) was analyzed with an analysis of covariance (ANCOVA) where the model included a start of treatment group (early/medium/late), and baseline MAS value. Other potential prognostic factors/covariates were tested for inclusion in the model in a stepwise selection process. The first step was based on univariate testing of candidate prognostic factors/covariates (full list provided in the Table e1). All factors with a critical significance level of 0.20 were included in the second step that compared each retained variable against the other retained variables (at the 0.001 level using Pearson correlation for continuous variables, Chi-square test/Fisher's exact test for categorical variables and Kruskal–Wallis for mixed categorical and continuous variables) to confirm that there was no strong link between them. If independence was not met for two variables (*p* < 0.001), the choice was done according to clinical relevance. Retained variables after step 2 were included in the stepwise multivariate model and kept if the *p* < 0.2. Patients categorized as medium-start were included in the model, but the primary comparison was between early-start and late-start.

Comparisons of (i) MAS_EWF_ at each study visit (Visits 2, 3, 4 and 5) and (ii) change in MAS_EWF_ scores at study Visit 5, between early-start and late-start patients (with and without stratification by previous BoNT exposure) were analyzed as secondary effectiveness variables using a similar model (ANCOVA including start-of-treatment group and baseline MAS) as the primary effectiveness endpoint. Other endpoints included descriptive analyses of MAS_EWF_ scores in the early, medium and late group with (exploratory) and without (secondary) stratification by BoNT exposure.

Between group differences in goal attainment and treatment satisfaction were analyzed using proportional odds models including treatment group as fixed effects. Changes in AROM, PROM and pain from Visit 1 to Visit 5 were analyzed using an ANCOVA where the model included start of treatment group and baseline values. Finally, changes from baseline in MAS and other endpoints, including EQ-5D-3L, were summarized descriptively by start of treatment group.

### Sample Size Estimation

It was estimated that a total of 150 patients was required to achieve 80% power in detecting an effect size of 0.5 on the composite MAS between the early-start and late-start groups at the 2-sided 5% significance level. To achieve a sample size of 150 patients in the early-start and late-start groups (75 in each group), a total of 300 patients was required.

## Results

### Patient Disposition and Baseline Characteristics

**Figure 1:**
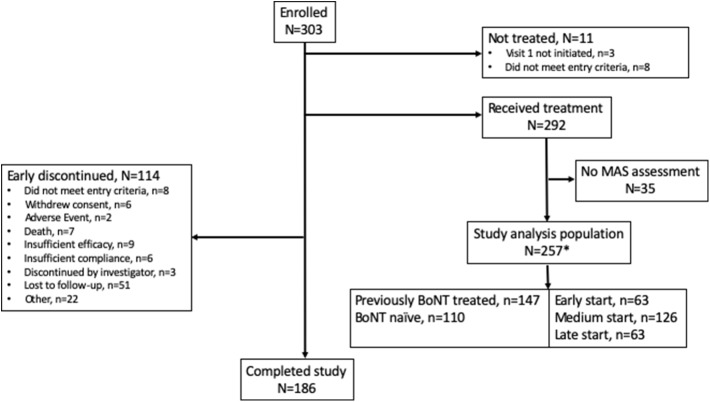
Patient disposition. *Five subjects in the study analysis population were not categorized in the start of treatment groups due to lack of information reported in the eCRF.

Of the 303 patients enrolled, 257 (84.8%) received treatment and had one post-baseline measurement of MAS, and 186 (61.4%) received 4 injection cycles and completed the study. The most common reason for early discontinuation was loss to follow-up (Figure 1). Per protocol, the study population was categorized into treatment groups: early-start *n* = 63, medium-start *n* = 126 and late-start *n* = 63; five patients were not categorized due to lack of information. Baseline characteristics are given in Table 1, overall 147 patients were previously-treated with a BoNT and 110 patients were naïve to BoNT treatment. Of note, the mean age at inclusion was higher and the mean age at stroke was lower in the late-start group vs. the other groups.

**Table 1:** Baseline (Visit 1) characteristics.

	**Early-start *N* = 63**	**Medium-start *N* = 126**	**Late-start *N* = 63**
Age (years); mean (*SD*)	59.70 (10.98)	60.58 (10.94)	62.25 (13.35)
Sex; *n*(%) male	41 (65.1)	89 (70.6)	30 (47.6)
Time since stroke event to first treatment (months); *N*, mean (*SD*) [95%CI]	*N* = 62 3.74 (1.75) [3.29, 4.18]	*N* = 126 20.11 (11.08) [18.16, 22.06]	*N* = 63 144.24 (90.85) [121.36, 167.12]
Time since arm spasticity onset to first treatment (months); N, mean (SD), [95%CI]	*N* = 59 2.60 (1.96) [2.09, 3.11]	*N* = 114 17.20 (11.76) [15.02, 19.38]	*N* = 59 138.63 (91.82) [114.70, 162.56]
Arm pattern; *n* (%)			
Type I	11 (17.5)	16 (13.2)	4 (6.3)
Type II	0	3 (2.5)	4 (6.3)
Type III	24 (38.1)	52 (43.0)	23 (36.5)
Type IV	27 (42.9)	40 (33.1)	30 (47.6)
Type V	1 (1.6)	10 (8.3)	2 (3.2)
Missing	0	5	0
MAS_EWF_ score^*^	4.82 (1.39)	4.53 (1.55)	4.83 (1.36)
Pain on VAS	3.92 (3.05)	2.80 (2.83)	2.30 (2.81)

All available data is presented, including the number of patients who had available data for each individual outcome.

* *Composite Modified Ashworth Scale (MAS) score = sum of elbow and wrist flexors (MAS_EWF_). VAS, visual analog scale*.

### Treatment Exposure

The mean ± SD time from stroke until start of first BoNT-A treatment was 3.74 ± 1.75 months in the early-start group, 20.11 ± 11.08 months in the medium-start group and 144.24 ± 90.85 in the late-start group. The time from documented onset of spasticity to start of first BoNT-A treatment was 1–5 months shorter than time since stroke; mean ± SD times since onset of spasticity were 2.60 ± 1.96, 17.20 ± 11.76, and 138.63 ± 91.82 months, respectively.

Most patients (*n* = 190, 73.9%) received 4 injections of abobotulinumtoxinA during the study period. Taken overall, the mean total dose of abobotulinumtoxinA over the study was 743.08 ± 356.60 U and the mean time between injections was 3.69 ± 1.27 months. Overall dose exposure per cycle by groups is presented in Table 2. Mean ± SD total doses increased over the course of the study; from 675.7 ± 308.6 U to 718.9 ± 473.8 U in the early-start group, and from 745.3 U ± 402.6 U to 861.9 U ± 401.6 U in the late-start group. The overall (averaged) time between study injections was longer in the early-start vs. late-start group (3.70 ± 1.16 months vs. 3.46 ± 0.76 months).

**Table 2:** AbobotulinumtoxinA exposure.

	**Early-start *N* = 63**	**Medium-start *N* = 126**	**Late-start *N* = 63**
Total dose (U) throughout study; Mean (SD) Median [range]	*N* = 63 719.32 (338.5) 645.0 [150.0–1833.7]	*N* = 125 714.23 (342.2) 655.0 [220.0–2112.5]	*N* = 62 807.45 (402.7) 780.0 [100.0–1800.0]
Time between study injections; (M) Mean (SD) Median [range]	*N* = 60 3.70 (1.2) 3.2 [2.1–7.4]	*N* = 125 3.78 (1.5) 3.3 [1.5–13.4]	*N* = 63 3.46 (0.8) 3.2 [2.7–6.1]
Length of exposure (days) Mean (SD) Median [range]	*N* = 63 375.3 (169.6) 387.0 [58.0–1018.0]	*N* = 126 402.9 (140.1) 381.5 [92.0–1113.0]	*N* = 63 390.6 (136.7) 386.0 [87.0–1029.0]

### Modified Ashworth Scale

**Figure 2:**
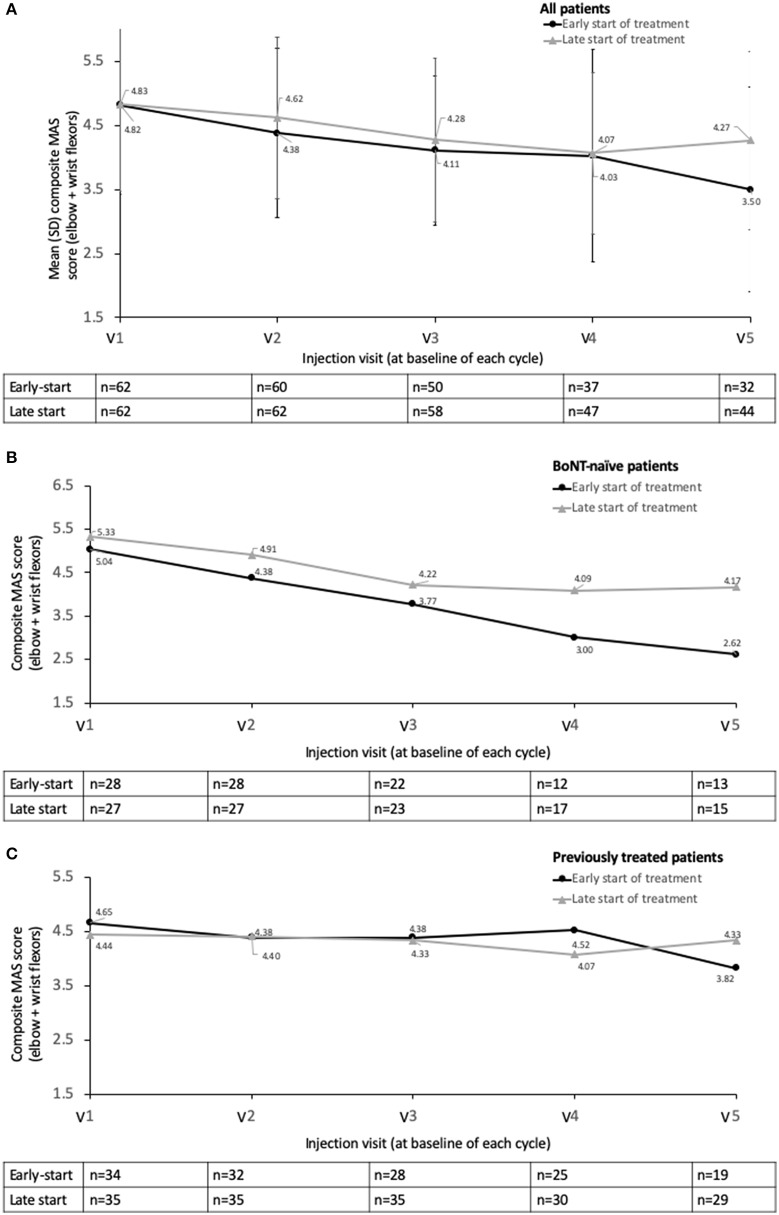
Descriptive statistics for MAS_EWF_ by study visit, early-start vs. delayed start subgroups **(A)** overall population, **(B)** BoNT-naïve population, and **(C)** previously treated population. Study visits were at end of treatment cycle.

Patients in all groups showed a reduction in MAS_EWF_ scores from baseline over the consecutive injection visits (i.e., at the end of each cycle) (Figure 2A). Although the primary analysis showed a numerically lower MAS_EWF_ score (LS mean) for the early- compared to the late- start treatment group (3.72 ± 0.28 vs. 3.87 ± 0.28), the difference at V5/last observed visit did not reach statistical significance (ANCOVA, *p* = 0.5465) (Table 3).

**Table 3:** Primary ANCOVA analysis.

	**Early-start group (*N* = 52)**	**Late-start group (*N* = 54)**
Least square mean (SE) MAS_EWF_ score	3.72 (0.28)	3.87 (0.28)
Difference in Least square means		0.15
95% Confidence interval for the difference		[−0.34, 0.64]
*p*-value		0.55

*The final ANCOVA model included the following covariates: start of treatment group, baseline MAS score, time since last injection before MAS assessment at V5 (days), spasticity pattern at baseline, overall achievement of individual treatment goals, concomitant therapy and average total dose (U)*.

Analysis by prior treatment showed that for the patients who were previously BoNT-naïve, there was a numerically larger reduction in the mean MAS_EWF_ scores in the early-start (despite a slightly lower baseline) vs. late-start patients from Visits 2 to 5 (Figure 2B); however differences were not statistically significant in the ANCOVA model (*p*-values ranged from 0.4788 to 0.8150). This clear pattern was not apparent in those patients who had been previously treated with a BoNT prior to study entry (Figure 2C). Previously treated patients showed lower MAS_EWF_ scores at baseline in both groups compared to BoNT-naïve patients.

### Goal Achievement

Analysis of baseline goal choice revealed that patients in the early-start group appeared more likely to list pain reduction as a key goal than those in the late-start group (54.0 vs. 39.7%, respectively) and functional improvement (38.1 vs. 27.0%, respectively). Conversely, improving ease of care and/or hygiene and supporting ease of PT and/or OT appeared to be chosen more frequently by patients with a longer duration of spasticity until BoNT treatment. Similar proportions of patients in all groups selected improvement of mobility as a treatment goal.

Overall at Visit 5, treatment goals were at least partially achieved for all groups (Table 4). At most visits, there were no significant differences in goal achievement between the early and late-start groups. However, at Visits 2 and 3, the treatment goal “functional improvement” was significantly better achieved in the late than in the early-start group (*p* = 0.0179 and 0.0312, respectively). At Visit 5, the treatment goals “Improvement of mobility/flexibility” and “Support and ease of PT/OT” were significantly better achieved in the early than in the late start of treatment group (both *p* = 0.04). Whereas, the mean number of hours per week for subjects using PT and/or OT decreased by about an hour in the early start of treatment group (from 3.49 ± 3.23 h at baseline to 2.34 ± 1.41 h at Visit 5), it increased by over an hour in the late start of treatment group (from 2.06 ± 1.37 h at baseline to 3.30 ± 7.08 h at Visit 5).

**Table 4:** Goal achievement.

**Goal type**	**Visit**	**Early-start**	**Medium-start**	**Late-start**
Improvement of mobility/flexibility; *n* (%)	**Visit 2** Fully achieved Partially achieved Not achieved Missing	8 (22.9%) 20 (57.1%) 7 (20.0%) 11	19 (22.1%) 58 (67.4%) 9 (10.5%) 8	7 (16.7%) 31 (73.8%) 4 (9.5%) 6
	**Visit 5** Fully achieved Partially achieved Not achieved Missing	9 (47.4%) 9 (47.4%) 1 (5.3%) 0	22 (34.4%) 38 (59.4%) 4 (6.3%) 4	6 (18.2%) 25 (75.8%) 2 (6.1%) 3
Pain reduction; *n* (%)	**Visit 2** Fully achieved Partially achieved Not achieved Missing	8 (32.0%) 14 (56.0%) 3 (12.0%) 8	16 (41.0%) 21 (53.8%) 2 (5.1%) 12	7 (33.3%) 11 (52.4%) 3 (14.3%) 4
	**Visit 5** Fully achieved Partially achieved Not achieved Missing	3 (37.5%) 4 (50.0%) 1 (12.5%) 1	9 (30.0%) 17 (56.7%) 4 (13.3%) 3	7 (38.9%) 9 (50.0%) 2 (11.1%) 2
Ease of care and hygiene; *n* (%)	**Visit 2** Fully achieved Partially achieved Not achieved Missing	8 (30.8%) 16 (61.5%) 2 (7.7%) 6	22 (38.6%) 30 (52.6%) 5 (8.8%) 9	13 (32.5%) 26 (65.0%) 1 (2.5%) 5
	**Visit 5** Fully achieved Partially achieved Not achieved Missing	9 (64.3%) 4 (28.6%) 1 (7.1%) 0	20 (45.5%) 22 (50.0%) 2 (4.5%) 2	15 (50.0%) 14 (46.7%) 1 (3.3%) 3
Support and ease of PT/OT; *n* (%)	**Visit 2** Fully achieved Partially achieved Not achieved Missing	9 (33.3%) 17 (63.0%) 1 (3.7%) 5	21 (35.0%) 37 (61.7%) 2 (3.3%) 6	9 (27.3%) 22 (66.7%) 2 (6.1%) 4
	**Visit 5** Fully achieved Partially achieved Not achieved Missing	12 (75.0%) 3 (18.8%) 1 (6.3%) 0	24 (50.0%) 23 (47.9%) 1 (2.1%) 2	11 (39.3%) 17 (60.7%) 0 1
Functional improvement; *n* (%)	**Visit 2** Fully achieved Partially achieved Not achieved Missing	2 (10.5%) 8 (42.1%) 9 (47.4%) 5	5 (12.5%) 29 (72.5%) 6 (15.0%) 8	1 (7.1%) 13 (92.9%) 0 3
	**Visit 5** Fully achieved Partially achieved Not achieved Missing	1 (9.1%) 5 (45.5%) 5 (45.5%) 0	2 (7.1%) 21 (75.0%) 5 (17.9%) 3	2 (16.7%) 7 (58.3%) 3 (25.0%) 1
Other; *n* (%)	**Visit 2** Fully achieved Partially achieved Not achieved Missing	0 2 (100.0%) 0 0	4 (66.7%) 1 (16.7%) 1 (16.7%) 0	1 (50.0%) 1 (50.0%) 0 0
	**Visit 5** Fully achieved Partially achieved Not achieved Missing	1 (100.0%) 0 0 0	3 (60.0%) 2 (40.0%) 0 0	2 (66.7%) 1 (33.3%) 0 0

### Pattern of Upper Limb Spasticity Involvement and Range of Motion

In terms of spasticity pattern, Types III and IV predominated at each visit. There were no significant differences at Visits 3 (*p* = 0.18) or 5 (*p* = 0.06) in the type of spasticity pattern between early-start and delayed-start groups.

Descriptive data for PROM and AROM at each visit are given in Table 5. The only significant difference between groups was PROM at the wrist joint at Visit 5, where the LS mean PROM was significantly higher in the early-start group vs. the late-start group (difference in LS mean −21.1 [95%CI: −38.7, −3.47], *p* = 0.02). Other changes in AROM and PROM at the wrist joint were not significantly different between groups.

**Table 5:** Passive and active range of motion by visit.

	**Early-start**	**Medium-start**	**Late-start**
**ELBOW**			
**PROM;** ***N*****, Mean (SD)**			
Visit 1	40 105.63 (40.16)	79 107.25 (37.86)	42 105.07 (34.53)
Visit 3	32 112.50 (39.72)	69 109.20 (35.04)	36 103.75 (35.68)
Visit 5	20 107.50 (36.58)	58 118.36 (38.22)	29 102.24 (38.44)
**AROM;** ***N*****, Mean (SD)**			
Visit 1	25 66.20 (41.91)	52 70.19 (41.79)	30 68.87 (39.25)
Visit 3	21 70.00 (38.57)	50 68.50 (39.96)	24 78.42 (33.93)
Visit 5	12 66.67 (32.64)	35 72.29 (44.58)	18 63.33 (39.33)
**WRIST**			
**PROM; N, Mean (SD)**			
Visit 1	43 88.07 (38.22)	87 84.74 (36.01)	46 88.65 (34.81)
Visit 3	33 97.88 (31.08)	76 93.49 (33.33)	32 91.88 (35.05)
Visit 5	24 110.83 (37.41)	60 103.25 (33.02)	30 95.00 (37.55)
**AROM;** ***N*****, Mean (SD)**			
Visit 1	30 46.33 (31.10)	53 45.28 (29.03)	24 43.50 (27.86)
Visit 3	21 41.90 (21.12)	44 43.64 (24.50)	18 46.50 (29.90)
Visit 5	19 49.21 (38.12)	35 45.29 (31.53)	17 50.24 (44.56)

### Pain

**Figure 3:**
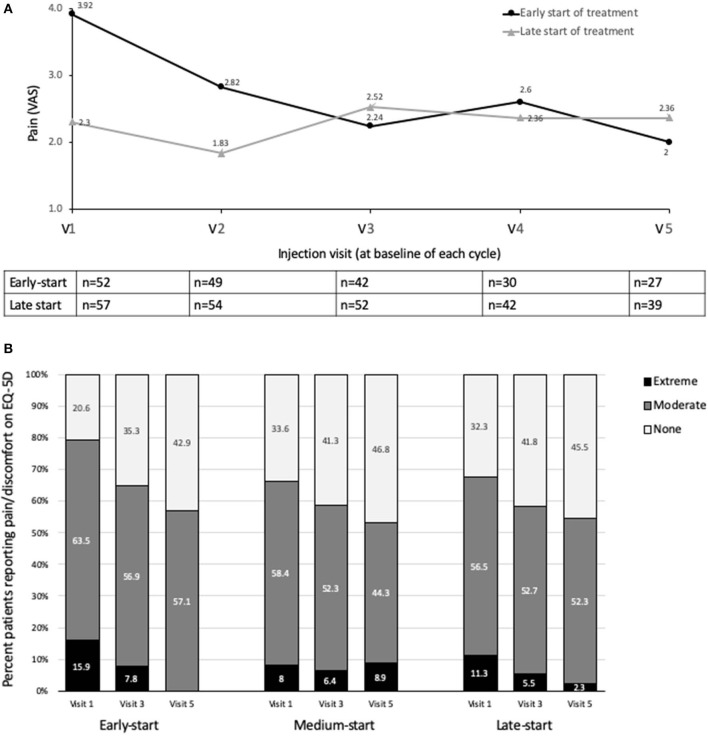
Effects on pain **(A)** Descriptive statistics for pain score (VAS), early-start vs. delayed start subgroups **(B)** Patient quality of life (EQ-5D pain/discomfort domain). Study visits were at end of treatment cycle. VAS, visual analog scale.

Patients in the early-start group reported higher pain scores than those in the late-start group at baseline (3.92 vs. 2.30, respectively). Whereas, patients in the early-start group showed a trend to reduced pain, and particularly over the first injection cycle, patients in the late-start group reported relatively stable pain scores over time (Figure 3A). However, while LS mean of pain scores tended to be lower in the early-start vs. late-start group from Visits 3 to 5, the differences were not significant in the ANCOVA model (*p*-value ranged from 0.055 to 0.196).

### Quality of Life and Treatment Satisfaction

Stronger increases in the mean quality of life EQ-5D index scores were observed in the early start of treatment group compared to the late start of treatment group. In the early-start group, mean EQ-5D index scores continuously increased from 0.54 ± 0.26 at baseline to 0.72 ± 0.18 at Visit 5. Although mean EQ-5D index scores in the late-start group also increased from 0.61 ± 0.31 at baseline to 0.65 ± 0.26 at Visit 5, the increase was not continuous. Overall, in all 5 dimensions, the percentage of subjects having no problems increased for all dimensions between Visit 1 and Visit 5 in the early-start group. By contrast, the percentage of subjects having no problems tended to remain similar in the late-start group (Figure e1). The main exception to this rule was pain, which tended to improve in all groups, and particularly in the early-start group. By Visit 5, no patient reported extreme pain in the early-start group (vs. 15.9% at visit 1) (Figure 3B).

**Figure 4:**
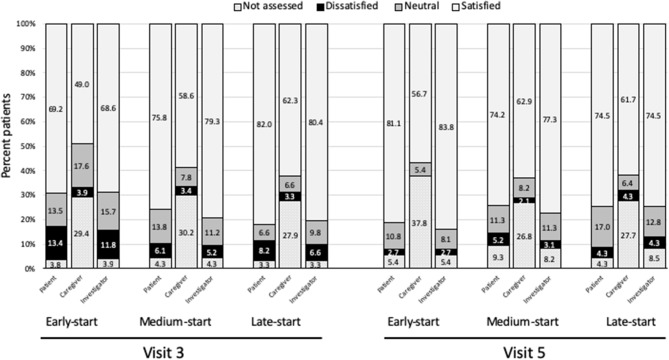
Satisfaction with treatment. Study visits were at end of treatment cycle. Satisfied = satisfied + very satisfied; Dissatisfied = unsatisfied + very unsatisfied.

Satisfaction with treatment was good across treatment groups; patients, investigators and caregivers were generally satisfied with the treatment at Visits 3 and 5 (Figure 4). There were generally no significant differences in treatment satisfaction between the early-start and delayed-start groups, except for the investigator's satisfaction at Visit 3 which was significantly better for the late-start group than for the early-start group (*p* = 0.047).

### Safety

No new safety issues arose from the study. A total of 47 AEs were reported, including 39 serious AEs in 21 patients. There were 7 deaths (myocardial infarction =1, cardiac arrest =1, cholangitis =1, lung cancer progression =1, cause not reported =3), none were considered treatment-related. Four of the 39 serious AEs were considered potentially related to treatment (listlessness, muscular weakness and two events of fall).

## Discussion

The results of this open-label, routine practice study did not show an overall significant difference in tone when abobotulinumtoxinA was started earlier (0–7 months) compared to later (36–443 months) in the patient treatment journey. Treatment with abobotulinumtoxinA was consistently effective in reducing spasticity as well as spasticity/stretch- related pain, whether started early after the stroke event or later, indicating a continued benefit of repeated abobotulinumtoxinA injections regardless of chronicity. MAS_EWF_ scores were, however, descriptively lower in the early-start group than the late-start group at each retreatment visit and at the end of study, and this trend was particularly apparent in patients who were new to BoNT-A treatment. No new safety findings emerged from this study with doses up to 2,000 U.

Clinical guidelines recommend that spasticity is treated when it becomes troublesome and impacts the patient's life (14). The similarity of baseline MAS scores between the three groups confirm prior observations that clinically relevant spasticity (as measured by muscle tone) develops in the first 3 months after stroke (10, 11, 18). Our definition of the “early-start” group generally aligns with the recently agreed definition of the “subacute phase” as proposed by The Stroke Recovery and Rehabilitation Roundtable taskforce (9). Most patients in the early-start group were either in the “early subacute” phase (1 week to 3 months) or the “late subacute” phase (3–6 months). Our findings show that patients treated in the subacute phase experience at least a similar (and a tendency for better) benefit than those treated in the chronic stages after stroke. Importantly, we observed continued effectiveness and safety with repeat treatments. Thus, as suggested by Rosales and colleagues (20), it follows that patients who receive early treatment will gain more time living with reduced spasticity than if they were treated later in their lifetime. In addition, the conditions for rehabilitation are typically better in the subacute vs. the chronic phase. There is evidence of continued neuroplasticity in the subacute phase, and it is intuitively easier to treat a patient before the development of intrinsic muscle changes and contractures that can worsen the severity of spasticity (9, 11, 28–30). Indeed, we saw a significant difference in PROM at the wrist joint between the early- and late- start groups. This is of direct practical importance because many of our patients were at risk of palmar flexion, which once the wrist goes beyond 70°, is hard to treat except by surgery. Further, it has been suggested that starting treatment early may *prevent* the development of secondary complications, allowing the spasticity to be effectively managed with lower doses of BoNT (18). Our findings support this concept of lower dosing in the subacute phase and also indicate that the time between injections may be longer in the earlier stages than the late stages.

The impact of previous treatment was highlighted by the descriptive results when analyzed by prior exposure to BoNT therapy. While there was a numerically larger reduction in mean MAS_EWF_ scores in the early-start vs. late-start BoNT-naïve patients, this pattern was not apparent in the previously-treated patients, again supporting the effectiveness of an early-start. Recent Phase III studies of repeat treatment with abobotulinumtoxinA have shown that spasticity parameters continue to improve with repeat treatments (31), and our observations in the treatment naïve patients suggest this may be especially true in the earlier (i.e., first three or four) treatment cycles where we saw a continual reduction in MAS scores–in both the early and late start groups. MAS scores for the previously treated patients were lower than for the BoNT-naïve group and were relatively stable, indicating that they were already well-managed. However, statistical significance between early- and late- start of treatment in the ANCOVA model was not achieved for BoNT-naïve patients, although this may also reflect the much reduced sample size. Another limitation is that, in line with its real-life design, we assessed MAS scores at end of treatment cycle, rather than at peak effect. It is likely that measuring the MAS and other parameters 3–4 months after injection when the pharmacological effect is expected to be waning, might hide a stronger effect of BoNT-A treatment *during* the treatment cycle.

Goal achievement was generally good in this study. Since treatment goals are necessarily tailored to be appropriate for the individual needs of the patient at the time of treatment, it is perhaps to be expected that there were no significant differences in goal achievement between the early- and late- start treatment groups. Of interest, patients in the early-start group reported higher pain scores and more frequently chose reduced pain as a treatment goal than those in the late-start group. This is noteworthy as pain in poststroke patients is often only associated with contractures and painful postures in chronic spasticity, which is less likely to be the cause of pain in the early-start group. This is an important observation as stretch-related pain is a common barrier to patient adherence with home-based physiotherapy (32). Previous studies have shown beneficial effects of BoNT-A on post-stroke pain (22, 33, 34), and our data extends this finding to patients with early post-stroke spasticity and particularly in the first abobotulinumtoxinA treatment cycle. The reasons for this better effect in the first cycle merit further exploration, but may include an indirect effect through reduction of painful spasms (33).

A common indication for BoNT-A therapy is to reduce tone in order to permit more effective OT and PT with respect to gaining function (26). While the goal of improving ease of PT or OT appeared to be more relevant for patients in the late-start group, it is pertinent to note that this goal was significantly better achieved in the early- than in the late-start of treatment group (*p* = 0.04). There is some limited evidence that certain task-based PT and OT approaches are more effective when started earlier post-stroke than later, and it may be that earlier use of BoNT-A may help patients make the most of an early window of opportunity (35, 36). Moreover, the number of hours spent at PT/OT reduced in the early compared to late group (mean decrease of almost 1 h vs. an increase of almost 1 h). It may be that BoNT-A injection (and study participation) caused some re-energization in late-start patients to participate in OT and PT programs. A limitation of this study is that we only considered hours of therapy, and not type of therapy. Other ongoing studies, such as the ULIS III program are currently collecting data to address this important issue (37).

Satisfaction with treatment was generally good across the whole patient cohort with few significant differences between groups. Ratings of treatment satisfaction were generally similar for patients, investigators and caregivers, although many caregivers were not assessed. This highlights the need for including the caregivers in discussing treatment expectations as well as providing caregiver support. Taken overall, we observed a generally stronger increase in quality of life scores in the early-start compared to the late-start group. In particular, patients in the early-start group showed good improvements in self-care and usual activities, whereas these domains remained more stable in the middle and late-start groups. Quality of life in terms of anxiety and depression domain scores improved in all patients during the study; here a limitation of this routine-practice study is that we cannot tease out the effects of the treatment from external factors such as acceptance and learning to cope with having spasticity. Other studies have found spasticity and social needs to have the strongest impact on quality of life following a stroke (38).

To our knowledge, this is the first prospective evaluation of the long-term effectiveness of routine botulinum toxin treatment on the recovery of upper limb spasticity in relation to the time since stroke. Limitations of the study include the high dropout rate primarily driven by loss to follow-up, with the consequence of relatively small patient numbers, especially at the later visits. As seen in the various analyses, prior exposure to BoNT therapy appears to be an important confounder of results. The study originally planned to primarily enroll BoNT naive patients, but problems with recruitment meant that the study had to be opened up to patients already under treatment. Since this was an observational study, we did not have complete datasets for each variable evaluated and it would have also been valuable to include more patient reported outcomes (as well as satisfaction with treatment) to give the patients perspective on their spasticity management. Finally, another important limitation is our quartile-based definition of early-start treatment, where the mean time since stroke was 3.2 months. This is just on the upper limits of the study-based definitions for “very early intervention” where botulinum toxin has been given within 2–12 weeks of the event to try and target neutrally mediated spasticity (18–20). Other factors having influenced the outcome might be the measurement not at peak effect, but rather at the end of the treatment effect and the shorter intervals and higher dose in the late compared to the early group. This is an interesting finding in itself, as it suggests similar or slightly better effects can be obtained when treating early–even when saving toxin and intervals.

## Conclusion

Taken overall, the results of this study confirm the utility of abobotulinumtoxinA injections at all stages of disease and support the idea that all patients whose spasticity is troublesome merit goal-directed treatment, regardless of whether it is started in the early or latter stages of the patients disease journey. Continuous treatment should be offered to patients where their treatment goals are considered amenable to BoNT-A treatment. Although our primary effectiveness analyses did not show a significant difference between early- and late- start of treatment, exploratory analyses in BoNT-naive patients showed a trend in favor of early treatment that merits further exploration.

## Data Availability Statement

Where patient data can be anonymized, Ipsen will share all individual participant data that underlie the results reported in this article with qualified researchers who provide a valid research question. Study documents, such as the study protocol and clinical study report, are not always available. Proposals should be submitted to DataSharing@Ipsen.com and will be assessed by a scientific review board. Data are available beginning 6 months and ending 5 years after publication; after this time, only raw data may be available.

## Ethics Statement

The studies involving human participants were reviewed and approved by the relevant independent ethics committee at each study center. The patients/participants provided their written informed consent to participate in this study. The study centers are as follows: Medizinische Universität Graz, Kantonale Ethikkommission Bern, Ordre National de Medicine, ISALA hospital, Universitätsklinikum Tübingen, Universitätsklinikum TU München, and Universitätsklinikum Düsseldorf.

## Author Contributions

JW and KF were involved in protocol development and wrote the first draft of the manuscript. JW, MH, MM, PG, NM, and KF were involved in patient recruitment and treatment. MK and PM were involved in data analysis. All authors contributed to the interpretation of results and approved the final version of the article.

### Conflict of Interest

Authors employed by Ipsen participated in designing the questionnaire and study; in analysis and interpretation of the data; and in review, approval of, and decision to submit the manuscript. JW has received honoraria for being on the Speakers' Bureau for Allergan, Ipsen, Merz, and for his role on advisory boards at Allergan, Ipsen, Merz. He also received an unrestricted research grant from Allergan and Merz. JH has received consultancy fees and speaker honorarium from Allergan, Ipsen, and Merz. He is also an investigator in an Ipsen study. HH has received consultancy fees and speaker honorarium from Allergan, Ipsen, and Merz. He is also an investigator in an Ipsen study. MH is an investigator in an Ipsen study. MK is employed by Ipsen. PM is employed by Ipsen. NM is an investigator in an Ipsen study. MM has received speaker honorarium from Ipsen and Merz. She is also an investigator in an Ipsen study. PG is an investigator in an Ipsen study. KF has received consultancy fees and speaker honorarium from Allergan, Ipsen, and Merz. He is also an investigator in an Ipsen study.
