# Supplementary material for: Effectiveness of AbobotulinumtoxinA in Post-stroke Upper Limb Spasticity in Relation to Timing of Treatment
Source: Front Neurol. 2020 Feb 28;11:104. doi: 10.3389/fneur.2020.00104 (PMC7058702; doi:10.3389/fneur.2020.00104)
Supplement: Supplementary file 1 [file Table_1.DOCX]

**Supplementary Appendix**

**Table e1. Potential prognostic factors/covariates included in the primary effectiveness ANCOVA model**

| **Potential prognostic factors/covariates** |
| --- |
| Time since last injection before MAS assessment at Visit 5/last study visit (continuous) |
| AS pattern at baseline (categorical, type I/type II/type III/type IV/type V) |
| Age at stroke (continuous) |
| Time since stroke (time between stroke and baseline) (continuous) |
| Duration of treatment (duration between first injection and last injection, continuous) |
| Average of overall total dose received during the study (continuous) |
| Concomitant therapies OT and/or PT (categorical, yes/no) |
| Controlled injection technique with electromyography (EMG) at Visit 4/last study visit (categorical, yes/no) |
| Country (categorical) |
| Pain in arm at baseline (continuous) |
| Overall EQ-5D index at baseline (continuous) |
| Active ROM Elbow at baseline (continuous) |
| Active ROM Wrist at baseline (continuous) |
| Passive ROM Elbow at baseline (continuous) |
| Passive ROM Wrist at baseline (continuous) |
| Overall achievement of individual treatment goal(s) according to investigator at Visit 5/last study visit (categorical, fully/partially/not achieved) |

**Figure e1. Patient quality of life, by EQ-5D domain**
